# Effect of Hot- and Cold-Water Treatment on Broccoli Bioactive Compounds, Oxidative Stress Parameters and Biological Effects of Their Extracts

**DOI:** 10.3390/plants12051135

**Published:** 2023-03-02

**Authors:** Ivana Šola, Dino Davosir, Emilie Kokić, Jana Zekirovski

**Affiliations:** Department of Biology, Faculty of Science, University of Zagreb, Horvatovac 102a, 10000 Zagreb, Croatia

**Keywords:** α-amylase, broccoli, α-glucosidase, climate change, hot- and cold-water treatment, hydrogen peroxide, kaempferol, lipase, proline, sinapic acid

## Abstract

The goal of this work was to define resistant and susceptible variables of young broccoli (*Brassica oleracea* L. convar. *botrytis* (L.) Alef. var. *cymosa* Duch.) plants treated with cold and hot water. Additionally, we wanted to single out variables that could potentially be used as biomarkers of cold/hot-water stress in broccoli. Hot water changed more variables (72%) of young broccoli than cold water (24%) treatment. Hot water increased the concentration of vitamin C for 33%, hydrogen peroxide for 10%, malondialdehyde for 28%, and proline for 147%. Extracts of broccoli stressed with hot water were significantly more efficient in the inhibition of α-glucosidase (65.85 ± 4.85% compared to 52.00 ± 5.16% of control plants), while those of cold-water-stressed broccoli were more efficient in the inhibition of α-amylase (19.85 ± 2.70% compared to 13.26 ± 2.36% of control plants). Total glucosinolates and soluble sugars were affected by hot and cold water in an opposite way, which is why they could be used as biomarkers of hot/cold-water stress in broccoli. The possibility of using temperature stress to grow broccoli enriched with compounds of interest to human health should be further investigated.

## 1. Introduction

Due to climate change, sudden and intense changes in weather conditions are becoming more frequent. Immobile organisms such as plants cannot “take refuge” from high or low temperatures but adapt their physiology to the new conditions to survive [1,2]. The intensity and direction of these changes depend on both the temperature and the plant species. Since adaptations at the metabolic level are fast, changes in the ambient temperature can be detected on the level of biochemical responses by which plants react to the new conditions [3,4,5]. Such changes are usually crucial yield-limiting factors for plants [6].

Studies conducted on various plant species, such as wheat, rice, corn, chili peppers, okra, tobacco and soybeans, have shown that heat stress affects plants in various, mostly negative, ways [7]. In corn, which was exposed to daytime temperature of 38 °C and nighttime temperature of 28 °C for 14 days, plasma, chloroplast and mitochondrial membranes were damaged [8]. In tobacco stored at a temperature of 43 °C for 2 h in the early stage of growth, a decrease in the net rate of photosynthesis as well as a decrease in antioxidant activity was observed [9]. In okra, at temperatures between 32 °C and 34 °C during growth, yield as well as the quality parameters of the legume, such as fiber content, decreased [10]. Additionally, high-temperature stress altered the expression of genes involved in the regulation of osmoprotectants, detoxifying enzymes, transporters and regulatory protein synthesis [11,12,13].

Plants that are sensitive to low temperatures can already suffer damage at temperatures close to 15 °C, while plants that are tolerant survive at temperatures slightly below 5 °C [14]. Cold stress induces different ultrastructural changes, primarily relating to membrane alterations [15]. Acclimatization to cold affects the composition of lipids in cells by increasing the proportion of unsaturated fatty acids that build phospholipids [16,17]. This is necessary to maintain the functionality of the plasma membrane. Plants adjust to low temperatures by synthesizing cryoprotective molecules such as soluble sugars, sugar alcohols and low-energy nitrogen compounds [18]. They decrease the activity of ATP synthase, inhibit Rubisco regeneration and photophosphorylation [19]. The tolerance of gene expression for improving adaptive capacity is also changed under cold stress [20].

Broccoli (*Brassica oleracea*) is rich in vitamins (C, K); β-carotene, a precursor of vitamin A; dietary fibers; polyphenols; fatty acids; minerals and glucosinolates—phytochemicals that are predominantly represented in *Brassica* vegetables [21]. These compounds contribute to the health benefits of broccoli, such as antioxidant, antiproliferative and antidiabetic properties and the protection of the cardiovascular system [22,23,24]. Temperature and irradiation have been recognized as the most important factors for the production of consumer-orientated quality broccoli [25]. Since broccoli is native to moderate climatic zones such as the Mediterranean region, high or low temperatures will cause perturbations in its phytochemical profile to survive. Such phytochemical perturbations might have consequences on the biological effects of a plant and its products [26].

As part of this work, we investigated the influence of hot and cold water on the metabolism of young broccoli (*Brassica oleracea* L. convar. *botrytis* (L.) Alef. var. *cymosa* Duch.) plants. Our aim was to (i) define the susceptible and resistant parameters of this plant during low- and high-temperature water stress, (ii) determine the degree of metabolism change of broccoli due to these two types of stress, and (iii) determine the degree of change in the biological effects of broccoli extracts due to the types of stress. The approach we chose was as follows: we spectrophotometrically determined the quantity of (a) total phenolics, flavonoids, flavonols, proanthocyanins, tannins, phenolic and hydroxycinnamic acids; (b) soluble sugars and total glucosinolates; (c) parameters of oxidative stress, hydrogen peroxide, proline and malondialdehyde and (d) photosynthetic pigments; (e) then, using the RP-HPLC method, we separated, tentatively identified and quantified individual phenolics and *L*-ascorbic acid in broccoli before and after in vitro simulated human digestion; (f) determined the antioxidant capacity; (g) measured the effect of broccoli extracts on the activity of the enzymes α-amylase, α-glucosidase and lipase and (h) statistically, using one-way analysis of variance (ANOVA), principal component analysis (PCA), hierarchical clustering and Pearson’s correlations, correlated all of the measured variables of broccoli in order to conclude which of the parameters, in what direction and at what intensity are affected by hot/cold-water treatment. The results showed that hot water changed more variables (72%) of young broccoli than cold-water (24%) treatment. Total glucosinolates and soluble sugars were affected by hot and cold water in opposite ways; therefore, they could be used as biomarkers of hot/cold-water stress in broccoli. Among the bioactive compounds and oxidative stress parameters, the variable that was most significantly affected was proline, which was 2.5 times higher after hot-water treatment. Among the variables presenting the biological activity of the extract, the most significantly affected was α-amylase inhibition, which was 1.5 times more inhibited by cold-water treatment than with the extract of the control group. Statistical analyses showed greater similarity between the control group and the group treated with cold water, while the group treated with hot water separated more significantly.

## 2. Results and Discussion

### 2.1. Effect of Hot- and Cold-Water Stress on Different Groups of Phenolic Compounds, Soluble Sugars and Glucosinolates in Broccoli

In order to survive, plants adapt their metabolism according to their environmental conditions. In our previous work, we showed that the phytochemical profile could even be changed intentionally to create plants enriched with the bioactive compounds of interest [27]. Since temperature is known to be one of the main modificators of the phytochemical composition of plants [28], in this study, we investigated the impact of hot- and cold-water stress on the broccoli metabolism. The amount of total phenolics, flavonoids, tannins and phenolic acids did not differ significantly between the experimental groups (Table 1). In the control group, total phenolics were recorded as 10.56 ± 0.55 mg gallic acid equivalents (GAE)/g dw, total flavonoids 16.31 ± 0.89 mg quercetin equivalents (QE)/g dw, total tannins 5.05 ± 0.12 mg catechin equivalents (CatE)/g dw and total phenolic acids 2.23 ± 0.36 caffeic acid equivalents (CAE)/g dw.

The proportion of hydroxycinnamic acids, as well as flavonols, significantly decreased in the group treated with hot water, the amount of hydroxycinnamic acids was 0.10 ± 0.01 mg caffeic acid equivalent/g dw and the amount of flavonols was 0.12 ± 0.01 mg QE/g dw. On the other hand, in the group treated with cold water it did not differ significantly compared to the control. This suggests that hydroxycinnamic acids and flavonols in broccoli are more sensitive to high-temperature water stress than low temperature. Regarding the low growing temperature, similar results as ours were recorded in spinach [29]; namely, not just that winter sweet treatment did not decrease flavonoid composition in spinach, but it even increased some of the components at certain timepoints.

Proanthocyanins, due to their astringent properties, allow the plant to defend against pathogens and predators. Additionally, due to their antioxidant potential, they also protect plants from abiotic stress [30,31,32,33]. The concentration of total proanthocyanins was the highest in the group treated with hot water, 1.21 ± 0.064 mg CatE/g dw. On the other hand, in the group treated with cold water, this value was not significantly different from the value of the control group. We hypothesize that broccoli might accumulate proanthocyanins by stimulation of their biosynthesis and/or inhibition of their catabolism as one of the acclimation mechanisms against hot-water stress. Contrary to our result, a higher proportion of proanthocyanins was recorded in the skin and seeds of the fruits of grapevines grown at low temperatures [34]. This indicates a specific response of different plant species at the level of total proanthocyanins to temperature stress.

Sugars are known as one of the main groups of osmoprotectants [35,36,37]. In our study, the amount of soluble sugars was significantly different between all three experimental groups. They were the highest in the group treated with hot water; 31.14 ± 1.47 mg sucrose equivalent (SucE)/g dw. Much like in our work, high-temperature stress caused an increase in sugar content in most genotypes of the *Vigna aconitifolia* species [38] and in lablab bean [39]. However, contrary to our expectation, in the group treated with cold water, the proportion was significantly lower than in the control group, 15.07 ± 1.61 mg SucE/g dw. The accumulation of soluble sugars is a common phenomenon during cold stress [40]. Therefore, this result is interesting and suggests the need for additional analyses. Soluble sugars help the plant adapt to temperature conditions by supporting the hardening of plant cell membranes and thereby preventing cell destruction. Such action by soluble sugars was described under conditions of cold shock [41]. Given that we did not completely freeze the plants, but only watered them with cold water, it is possible that the shock was insufficient to elicit the biosynthesis of soluble sugars.

Glucosinolates and their hydrolysis products play a key role in the growth and development of plants, their taste and their defense systems [42,43,44]. In our work on young broccoli, we recorded significant changes in the proportion of total glucosinolates after both treatments. Cold water significantly increased the proportion of these compounds, 34.96 ± 2.37 mg sinigrin equivalent (SinE)/g dw. On the contrary, hot water decreased their amount to the value of 24.07 ± 1.34 mg SinE/g dw. This indicated a high degree of susceptibility of broccoli glucosinolates to temperature stress, and the specificity of the response depending on temperature. These results also suggest the potential to use low temperatures to grow broccoli enriched with glucosinolates. Similarly to our study, the amount of all types of glucosinolates decreased with increasing temperature in red cabbage [45]. Likewise, low temperature increased the concentration of total glucosinolates in kale [46,47].

### 2.2. Effect of Hot- and Cold-Water Stress on Parameters of Oxidative Stress and Pigments in Broccoli

A direct result of stress-induced cellular changes is the overproduction of highly reactive and toxic oxygen species (ROS) that damage proteins, lipids and carbohydrates and cause oxidative stress [48]. In our work, both types of stress significantly affected the amount of hydrogen peroxide in broccoli plants; however, this occurred in an opposite way (Table 2). More precisely, hot-water treatment significantly increased the concentration of hydrogen peroxide from 2.33 ± 0.13 µM/g dw in the control group to 2.56 ± 0.30 µM/g dw. Cold water decreased it to the concentration of 1.93 ± 0.12 µM/g dw. Proline was affected in the same way, as hot water significantly increased the concentration to 3.31 ± 0.09 µM/g dw, which was almost 2.5 times higher than in the control group. Cold water decreased it to 1.15 ± 0.12 µM/g dw. This result suggests that hydrogen peroxide and proline can be used as biomarkers of hot/cold-water stress in young broccoli. Lipid peroxidation, that is, malondialdehyde concentration, was affected by hot-water treatment only and was increased from 23.92 ± 0.74 µM/g dw in the control group to a concentration of 30.62 ± 1.39 µM/g dw. When we look at all three parameters of oxidative stress together, we notice that the most significant change occurred in the group treated with hot water. This indicated the highest level of oxidative stress induction in plants treated with hot water, which occurred on the level of proline. Similar to our result, an increased level of proline under high-temperature stress was recorded in lablab bean (Dolichos lablab) [39].

If the share of photosynthetic pigments in a plant is significantly changed, the possibility of using solar energy for growth will be changed, and thus the development and functioning of the plant will also change. In our work, the values of all pigments, except for chlorophyll *a*, were significantly different between the group treated with hot water and the remaining two groups. The treatment with hot and cold water did not significantly affect the concentration of chlorophyll *a* in young broccoli plants, but hot water significantly increased the concentration of chlorophyll *b*, raising it to 5.35 ± 0.34 mg/g dw. We recorded the same results for lycopene and porphyrins; hot water significantly increased their concentration (0.28 ± 0.016 mg lycopene/g dw, and 28.27 ± 1.09 mg porphyrins/g dw), while cold water did not affect it. A similar result with drought treatment was also previously recorded in young Chinese cabbage plants [49]. Likewise, an increase in the proportion of chlorophyll *b* was also recorded in rice genotypes that were highly resistant to high-temperature stress [50]. It is assumed that a high proportion of chlorophyll *b* is associated with high temperature tolerance. Based on our results, we hypothesize that photosynthetic pigments in young broccoli plants, except for chlorophyll *a*, are more susceptible to high- than to low-temperature stress. Given that chlorophyll *a* is the primary pigment of photosynthesis, the stability of this structure in different environmental conditions is extremely important for plant survival. This was probably the reason why its share in broccoli did not change due to temperature stress. A different result was recorded in cucumber seedlings [51]. More precisely, cucumber seedlings were treated with low (7 °C) and high (42 °C) temperatures, and in both cases a decrease in the proportion of total chlorophyll was recorded. However, a more significant decrease in the proportion occurring with low- rather than high-temperature treatment was recorded.

It is interesting to note that in the case of the concentration of total carotenoids, the situation was exactly the opposite. Hot water significantly lowered their concentration to was 0.40 ± 0.19 mg/g dw only, while there was no significant difference between the control group and the group treated with cold water. The same result was recorded with different genotypes of rice [50] and wheat [52]. When we compare the treatments in our study, similarly to chlorophyll, lycopene and porphyrins, hot-water treatment had a more significant effect on total carotenoids than cold-water treatment. The reason why hot water decreased carotenoids might be the price paid for enhanced chlorophyll, lycopene and total porpyhrin synthesis. Chlorophyll is obviously more important in the defense of young broccoli against hot water than total carotenoids. Additionally, young broccoli is less resistant to hot than to cold water.

### 2.3. Effect of Hot- and Cold-Water Stress on the Concentration of Individual Phenolic Compounds and Vitamin C in Broccoli

Both types of temperature stress significantly increased the concentration of *p*-coumaric acid compared to the control group (Table 3). Treatment with hot water caused a more intense increase in the concentration of this acid than treatment with cold water. Specifically, a concentration of 14.22 ± 0.68 mg/g dw was recorded in the control group, 16.72 ± 0.51 mg/g dw in the group treated with cold water and 19.04 ± 0.29 mg/g dw in the group treated with hot water. Previous research has shown a differential accumulation of phenolic compounds in plants depending on the type of abiotic stress [53]. Given that we applied two different types of stress, we also expected different concentrations of phenolic compounds depending on whether the broccoli was treated with hot or cold water. *p*-coumaric acid is important for the plant organism because it is a precursor of polyphenolic compounds, such as flavonoids and other phenolic acids, as well as lignin [54], a compound in plant cell walls that contributes to their strength and protection from environmental conditions. We assume that this is one of the possible reasons why both types of stress led to an increase in the concentration of *p*-coumaric acid; to supply the plant with the main precursor of a series of defense compounds. The same result was also recorded in Chinese cabbage [55], different wheat cultivars exposed to heat stress [56], tomato [57] and the genotype Predator of the *Festuca trachyphylla* species [58]. However, contrary to our results, in the leaves of grapevines that were subjected to low-temperature stress for a week, a decrease in the concentration of *p*-coumaric acid was recorded [59]. This suggests the specificity of the defense response mediated by *p*-coumaric acid in different plant species. For *p*-coumaric acid, the ability to alleviate the symptoms of diabetes has been proven [60,61,62] through various mechanisms. The fact that cold-water treatment significantly increased the concentration of this acid suggests a great potential of the application of this type of stress for the cultivation of broccoli with increased health value.

The concentration of ferulic acid in broccoli was not significantly affected by cold-water stress, but hot-water stress significantly increased its concentration to 64.99 ± 2.62 mg/g dw. In the control group and the group treated with cold water, the value was 49.41 ± 4.80 mg/g dw and 43.18 ± 3.28 mg/g dw, respectively. Recently, it was reported that high-temperature stress did not significantly affect the concentration of this component in wheat [56]. A decrease in the concentration of ferulic acid in grapevine leaves treated with low temperature was recorded [59]. On the other hand, in tomato seedlings [57] and Chinese cabbage [55], an increase in the concentration of ferulic acid was recorded after low-temperature stress. This points to a specific response of different species to temperature stress at the level of this phenolic acid. Such differences in results can be attributed to differences in the intensity and duration of stress, the stage of plant development and the part of the plant analyzed, e.g., flower, fruit, leaf, stem and root [56].

Contrary to the previous one, sinapic acid in broccoli was extremely sensitive to stress caused by hot water and its concentration decreased almost by half, to 339.80 ± 37.67 mg/g dw. Cold-water treatment had no significant effect. For comparison, an increase in the concentration of this acid in Chinese cabbage treated with cold stress was recently recorded [55], and no significant change was observed in the concentration of this acid in wheat grown at elevated temperatures [56]. This suggests the importance of plant matrices for the stability/susceptibility of a bioactive compound under the impact of environmental stress.

When we compare all three identified phenolic acids, we can see that *p*-coumaric acid in young broccoli is sensitive to both types of stress, while ferulic and sinapic acid are susceptible to hot-water treatment only. Hot water affected all three phenolic acids with a very similar intensity: change in a concentration between 32% and 36%. However, *p*-coumaric and ferulic acid concentration was increased at the expense of sinapic acid, whose concentration was decreased. Based on this, we conclude that under hot-water stress, young broccoli will synthesize more intensively phenolic acids containing two hydroxyl groups only (*p*-coumaric acid), or two hydroxyl groups and one methoxy group (ferulic acid). Phenolic acid containing two hydroxyl and two methoxy groups (sinapic acid) will be decreased, probably because two methoxy groups sterically hinder the antioxidant potential of hydroxyl groups and hence reduce the functionality of sinapic acid in plant defense against ROS. From another point of view, as with a study on durum wheat cultivars [56], hot-water treatment increased the minor identified phenolic acids, i.e., *p*-coumaric and ferulic. The fact that cold-water stress affected *p*-coumaric acid only suggests that for young broccoli, cold water is less stressful than hot water on the level of these phenolic acids. Additionally, half the increase than that of hot-water treatment of an acid containing two hydroxyl groups, without any methoxy group, was enough to protect broccoli from cold-water stress.

The flavonols we tentatively identified in broccoli using the RP-HPLC method were kaempferol and quercetin. Kaempferol was the predominant flavonol in young broccoli. Its concentration was almost three times higher than that of quercetin. The treatment with cold water did not significantly affect the concentration of kaempferol; however, treatment with hot water led to a significant decrease in its concentration. Based on this, we assume that the broccoli matrix “protects” kaempferol better from cold stress than from elevated temperatures. On the other hand, in tomato seedlings treated with low and high temperatures, the concentration of kaempferol increased [57]. We hypothesize that in young broccoli some other phytochemicals are harnessed to defend it against hot-water treatment, and not kaempferol. One of these could be quercetin.

The concentration of quercetin in broccoli increased significantly under the influence of hot water, to 35.11 ± 0.73 mg/g dw, compared to 32.93 ± 0.49 in the control group. Cold water did not show a significant effect. For comparison, in treated tomato seedlings, the concentration of quercetin increased after both low- and high-temperature treatment [45]. Given that high-temperature water stress in broccoli caused an increase of quercetin and a decrease of kaempferol, we assume that quercetin plays a more significant role in the defense of this species against high temperature, although it was present in a significantly lower concentration than kaempferol. This is a good example that the quantity is not crucial, but the chemical structure, i.e., the mode of action in the cells. Obviously, hot-water treatment supports flavonols with higher number of hydroxyl groups instead of those with less. Additionally, based on this result, we assume that quercetin and kaempferol in broccoli are in an antagonistic relationship with each other during heat stress. Similar relationship between these two flavonols we had previously recorded in *Crocus* varieties grown at different altitudes [63]. Namely, quercetin:kaempferol ratio was higher in varieties collected at mountains where the UVB radiation was more intense and has a stronger effect on plants. Therefore, we conclude that in a situation of a significant stress, plants will increase the biosynthesis of a flavonol containing more hydroxyl groups to better scavenge reactive oxygen species (ROS).

Vitamin C (*L*-ascorbic acid) is one of the most important parameters of the nutritional quality of food plants and is an essential nutrient for the human body [64,65,66]. The concentration of vitamin C increased significantly in broccoli treated with hot water to 1522.99 ± 70.30 mg/g dw. In the group treated with cold water, there was no significant change compared to the control: 1143.09 ± 33.32 mg/g dw and 1144.43 ± 66.64 mg/g dw, respectively. As in our work, in the species *Festuca arundinacea* Schreb [66], tall fescue [67] and in lablab bean [39], the concentration of vitamin C increased significantly in parallel with the increase in temperature. In the work carried out on citrus fruits, which are the richest in vitamin C, it was shown that the temperature of fruit processing significantly affected the concentration of vitamin C. Thus, the concentration of vitamin C was higher in juice squeezed at a temperature of 20 °C than at a temperature of 80 °C [68]. This indicates the thermolability of vitamin C during the processing of plant material and, more importantly, the completely different effect of high temperature on this vitamin in intact living plant and harvested plant material. It might be that the vitamin C in a living cell is to important to be destroyed under high-temperature stress, and therefore it is increased at the cost of some other phytochemicals.

### 2.4. Concentration of Individual Phenolic Compounds and Vitamin C during In Vitro Simulated Digestion of Broccoli: Comparison of Control, Hot-Water- and Cold-Water-Stressed Plants

**Figure 1:**
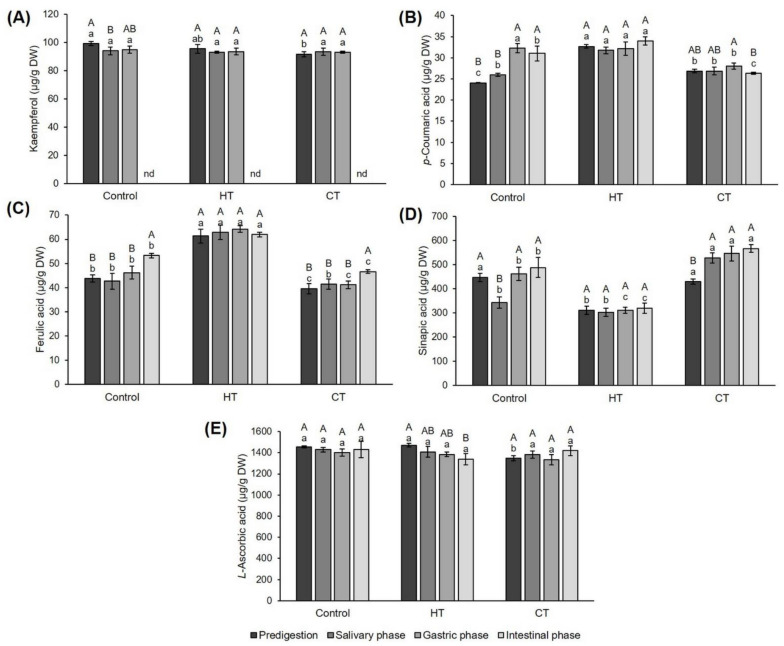
Effects of hot- and cold-water stress on the concentration of individual phenolic compounds: (**A**) kaempferol, (**B**) *p-*coumaric acid, (**C**) ferulic acid, (**D**) sinapic acid and (**E**) vitamin C from broccoli during in vitro simulated human digestion. The results are expressed in μg/g of dry weight. Values represent mean ± standard deviation of three biological replicates. Different capital letters indicate a significant difference among the values of digestion phases for one sample, and different small letters indicate a significant difference among the values of three samples in one digestion phase (ANOVA, Duncan test, *p* ≤ 0.05). Con = plants watered with room-temperature water, HT = plants watered with high-temperature water, CT = plants watered with cold water.

Due to the interaction of the extract matrix with gastrointestinal enzymes and bacteria in the human digestive system, the concentration of bioactive compounds varies between the phases of digestion [69,70]. Since the composition of the plant matrix depends on the growing conditions, we analyzed whether hot- and cold-water stress affected the bioactive compound concentration in the salivary, gastric and intestinal phases of digestion using an in vitro model. Flavonol kaempferol concentration in control plants was decreased after the salivary phase of digestion, while hot- and cold-water stressed plants did not show any differences in this compound concentration during digestion (Figure 1A). *p*-coumaric acid in control plants was increased after the gastric and intestinal phase, while in hot- and cold-water-stressed plants there was no change in its concentration during in vitro digestion (Figure 1B). Treatment with hot water increased the concentration of this acid in each of the digestion phases except the gastric one, where it did not differ compared to the control group of plants. Based on this result, we concluded that hot-water-stressed broccoli presents a better source of *p*-coumaric acid in the human digestion system than broccoli treated with room-temperature water. Ferulic acid concentration was increased after the intestinal phase in control and cold-water-stressed plants, while it was not changed in hot-water-stressed ones (Figure 1C). Same as with *p*-coumaric acid, hot-water-stressed broccoli presented a better source of ferulic acid in the human digestion system than broccoli treated with room-temperature water. Sinapic acid in control plants was decreased after the salivary phase of digestion, while in cold-water-stressed plants it was increased in each of the digestion phases (Figure 1D). When we compare the level of sinapic acid for each digestion phase between the groups of plants, we can see that hot/cold water oppositely affected its concentration to that of *p*-coumaric and ferulic acid. Namely, *L*-ascorbic acid (vitamin C) concentration was not changed during the in vitro digestion of control and cold-water-stressed plants. However, it decreased after the intestinal phase of hot-water-stressed plants (Figure 1E). By comparing samples within the digestion phase, no differences could be detected. This is interesting because in the original extract, the concentration of vitamin C was significantly higher in hot-water-stressed broccoli (Table 3). This means that during digestion, the matrix of hot-water-stressed broccoli cannot “protect” this vitamin from being degraded.

Along with the concentration of identified compounds, we also calculated the bioavailability of these compounds (Supplementary Table S1). In control plants, the bioavailability of *p*-coumaric and sinapic acid was higher after the gastric and intestinal phases of in vitro digestion and did not differ between those two phases. Ferulic acid was more bioavailable after the intestinal phase, while salivary and gastric did not differ between each other. Kaempferol availability did not differ among the salivary and gastric phases and could not be detected after the intestinal phase of digestion at all. Bioavailability of vitamin C did not differ between any of the phases. In cold-water-stressed plants, *p*-coumaric acid was more bioavailable in the gastric than intestinal phase; salivary phase bioavailability did not differ during either of the remaining phases. Ferulic acid was the most bioavailable in the intestinal phase, while sinapic acid, kaempferol and vitamin C bioavailability did not differ between the phases. Kaempferol was under the detection limit in the intestinal phase. In hot-water-stressed plants, we recorded no difference between the digestion phases for any of the identified compounds. When we compare the bioavailability of each of the compounds in each of the digestion phases between the control and tested plants, we can see that *p*-coumaric acid bioavailability in the salivary phase decreased after both types of stress. Ferulic acid bioavailability was not affected by cold-water stress in any of the phases, while hot-water stress decreased its availability in the intestinal phase. On the contrary, sinapic acid availability was increased after cold-water treatment in every digestion phase. Hot-water stress increased its availability in the salivary phase only, while availability in other phases was not changed. Bioavailability of kaempferol was affected by cold-water stress only; it was increased in the salivary and gastric phase. Hot-water stress did not significantly change kaempferol bioavailability. Interestingly, vitamin C bioavailability was not significantly affected by any type stress in any of the digestion phases.

### 2.5. Effect of Hot- and Cold-Water Stress on Antioxidant Potential of Broccoli Extracts

Oxidative stress is a common response of plants exposed to extreme temperatures [71,72]. ROS were recognized as ubiquitous markers of oxidative stress and signaling events for the induction of adaptive stress responses [73]. During temperature stress, the increased production of ROS is a major risk factor for plant cells, and therefore it is crucial that the concentration of ROS-detoxifying compounds also increases [74]. Although almost all organisms possess antioxidants and several enzyme systems such as superoxide dismutase, catalase, glutathione peroxidase and glutathione reductase to protect them against oxidative damage, these systems cannot completely defend them from damage. Therefore, antioxidants or foods containing high concentrations of antioxidants are needed to help scavenge free radicals and reduce oxidative damage. The currently available synthetic antioxidants, including butylated hydroxytoluene (BHT) and butylated hydroxyanisole (BHA), have many unwanted side effects on human health [75], which limits their application. Therefore, the trend to replace them with natural antioxidants that are physiologically easier to tolerate has gained importance. Given that there are different types of antioxidants in plant cells, we used three different methods of measuring antioxidant potential, ABTS, DPPH and FRAP, to obtain the most reliable information. ABTS, unlike DPPH and FRAP, is soluble in both aqueous and organic media, which makes it more suitable for the analysis of hydrophilic and lipophilic antioxidants and pigments [76]. Therefore, we expected to record a difference compared to the control group primarily with this method, but this was not the case. None of the treatments significantly changed the antioxidant potential measured by the ABTS and FRAP methods, but both caused a decrease in the potential measured by the DPPH method (Table 4). This difference in results between different methods was due to the different types of antioxidants present in the samples that react differently with the radicals used. A similar result, related to the suitability of the methods, was recorded when measuring the antioxidant potential of the mycelium of different types of mushrooms [75]. Based on these results, we conclude that the use of low- or high-temperature water cannot significantly improve the antioxidant potential of broccoli. Even more so, according to the DPPH method, this potential was reduced. A similar result has already been recorded on *Brassica oleracea* vegetables; in the *acephala* (kale) group, the ABTS method showed a lowering of the antioxidant potential, while in the *capitata* (cabbage) group, there was no significant impact [77]. Likewise, in Chinese cabbage treated with low temperature, the antioxidant potential measured by the DPPH and ORAC method, as well as the activity of the key antioxidant enzymes catalase and peroxidase, decreased [55].

### 2.6. Effect of Hot- and Cold-Water Stress on the Potential of Extracts of Young Broccoli Plants to Inhibit α-Amylase, α-Glucosidase and Lipase Enzyme

The search for natural inhibitors of α-amylase, α-glucosidase and lipase enzyme is becoming more intense because synthesized drugs have unwanted side effects [78,79]. Many fruits have been shown to contain inhibitors of these enzymes [28,80,81,82]; however, there is much less data for the inhibitory potential of vegetables [83,84].

**Figure 2:**
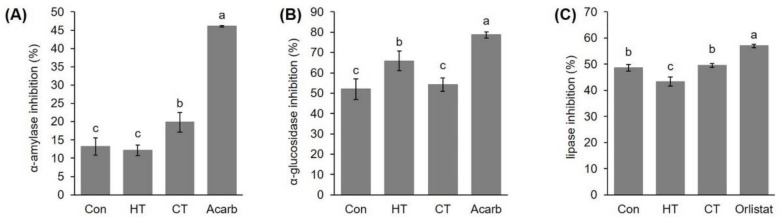
Effects of hot- and cold-water stress on the potential of extracts of young broccoli plants to inhibit the enzymes (**A**) α-amylase, (**B**) α-glucosidase and (**C**) lipase activity expressed in percentage of inhibition (%). Values represent mean ± standard deviation of three biological replicates. Different letters indicate a significant difference among the values (ANOVA, Duncan test, *p* ≤ 0.05). Con = plants watered with room temperature water, HT = plants watered with high temperature water, CT = plants watered with cold water, Acarb = acarbose.

Enzyme α-amylase participates in the hydrolysis of starch and glycogen, and its inhibition is an approach for carbohydrate intake disorders treatment, such as diabetes and obesity, as well as dental caries and periodontal disease [85]. Plants are a great, renewable source of chemical compounds with the potential to inhibit α-amylase and can be used as therapeutic or functional foods [86,87]. So far, about 800 plant species with antidiabetic properties have been recorded [85]. Among others, extracts of lyophilized radish sprouts (*Raphanus sativus* cv. Rambo) inhibited α-amylase activity [87]. Moreover, extracts of two broccoli varieties, *Brassica oleracea* L. convar. *Italica botrytis* (L.) Alef. var. *cymosa* Duch. and *Brassica oleracea acephala* L. convar. *acephala* (DC.) Alef. var. *sabellica* L. showed the ability to inhibit α-amylase activity [88]. In our work, the group treated with cold water inhibited the activity of this enzyme significantly more, achieving a level of inhibition of 19.85 ± 2.70%, than the control group and the group treated with hot water (Figure 2A). The α-amylase inhibition ability of the group treated with hot water and the control group was not significantly different: 12.11 ± 1.68% and 13.26 ± 2.36%, respectively. Neither plant group was more efficient in the inhibition of α-amylase than the standard inhibitor acarbose with a concentration of 20 mg/mL, which inhibited 46.07 ± 0.19% of enzyme activity. Considering our results, if one of the goals of broccoli consumption would be an improved inhibition of α-amylase, it would certainly be reasonable to treat broccoli with cold water during cultivation. This result also suggests further research into the possibility of using cold-water stress to increase the antidiabetic properties of plant species.

Inhibition of α-glucosidase activity is another possibility for regulating blood glucose level. Different types of α-glucosidase inhibitors have been found in microorganisms and plants, including alkaloids, phenolics, curcuminoids and terpenoids [28,89,90]. Regarding the α-glucosidase activity in our study, hot-water treatment significantly increased the inhibition potential of broccoli extracts to 65.85 ± 4.85%, compared to 52.00 ± 5.16% in control plants (Figure 2B). Cold-water treatment did not significantly affect the inhibition potential of extracts toward this enzyme, which reached 54.26 ± 3.24%. As in the case of α-amlyase, the standard compound, acarbose, showed the highest inhibition potential, 78.69 ± 1.47%.

Obesity is a strong risk factor for diseases such as hypertension, arteriosclerosis and diabetes [91]. One of the ways to prevent obesity is the inhibition of fat absorption from the intestines, that is, inhibition of pancreatic lipase which breaks down triglycerides into free fatty acids and glycerol. Only a small number of substances, such as orlistat (tetrahydrolipstatin), inhibit the activity of lipase [92]; however, such substances cause unpleasant side effects in the gastrointestinal system and kidneys and, in addition, are expensive. It is known that many plant species inhibit the activity of lipases thanks to the presence of specialized metabolites such as polyphenols, which are strong inhibitors of pancreatic lipase [93]. Recently, the potential of methanolic extracts of twelve medicinal plant species and four types of agricultural waste to inhibit lipase activity was analyzed [94]. The results showed that methanol extracts of white poplar twigs (*Populus alba),* green *Ononis vaginalis* and *Asparagus stipularis* have excellent inhibitory ability: 98%, 94% and 92%, respectively. For comparison, the standard orlistat with a concentration of 1 μg/mL showed 90% inhibition. Further analyses revealed that the flavonoids taxifolin and ampelopsin were the strongest inhibitors, followed by *p*-hydroxybenzoic acid. To the best of our knowledge, there are no published data about the influence of temperature stress on the ability of plant extracts to inhibit lipase. Therefore, as part of our work, we investigated whether temperature stress would change the phytochemical composition of a plant to such an extent that the ability of its extract to inhibit lipase would also significantly change. The results showed that treatment with hot water significantly reduced the ability of broccoli extract to inhibit lipase activity; the inhibition level was 43.32 ± 1.75% (Figure 2C). In the group treated with cold water, the ability to inhibit lipase did not differ significantly compared to the control group; it was 49.54 ± 0.80% and 48.58 ± 1.34%, respectively. Additionally, the inhibition values were relatively close to the inhibition percentage of the orlistat standard at a concentration of 20 mg/mL, 56.96 ± 0.72%. This indicates the potential of broccoli for this purpose and suggests further research in this direction. This result also suggests that high temperature has a negative effect on broccoli compounds with inhibitory activity toward lipase, and if one wants to preserve this potential, broccoli should not be exposed to high temperatures.

### 2.7. Representation and Distribution of Decreased, Unchanged and Increased Variables in Young Broccoli Plants Due to Hot/Cold-Water Treatment

Based on all the measured variables, we concluded that hot water changed more variables of young broccoli than cold-water treatment. To be precise, 72% of measured variables were changed after hot-water-, and 24% after cold-water treatment (Supplementary Figure S1, Supplementary Table S2). Among the changed variables upon hot-water treatment, 62% of them increased and 38% decreased, while upon cold-water stress, 43% increased and 57% decreased.

Regarding the identified individual compounds, the order of compounds in each group is the same; the most represented was *L*-ascorbic acid with 56% in the control group, 57% in the group treated with water of reduced temperature and 72% in the group treated with water at an elevated temperature (Supplementary Figure S2). The second most abundant was sinapic acid; however, unlike *L*-ascorbic acid, its concentration in the treated groups was lower than in the control group. Specifically, it was 31% in the control group, 30% in the group treated with cold water and only 16% in the group treated with hot water. The third most abundant compound was kaempferol, followed by ferulic acid, quercetin and, finally, *p*-coumaric acid.

Some of the variables were affected by both types of stress in the same direction, and some in the opposite direction. For example, total flavonols, proanthocyanins, phenolic acids, hydroxycinnamic acids, soluble sugars, glucosinolates, ferulic and sinapic acid, kaempferol, hydrogen peroxide, proline, malondialdehyde, α-amylase and lipase activity were affected oppositely by hot- and cold-water treatment (Supplementary Figures S3 and S4). Among the bioactive compounds and oxidative stress parameters, the variable that was most significantly affected was proline. It was 2.5 times higher after hot-water treatment (Supplementary Figure S4B). Among the variables presenting the biological activity of the extract, the most significantly affected was α-amylase inhibition. With cold-water-treatment it was 1.5 times more inhibited than with the control group extract (Supplementary Figure S4C). The resistable variables in young broccoli that were not changed by either of the stress types were chlorophyll *a*, total phenolics, flavonoids, tannins and phenolic acids, as well as antioxidant potential measured by the ABTS and FRAP methods.

### 2.8. Chemometric Data Analysis

#### 2.8.1. Principal Component Analysis

**Figure 3:**
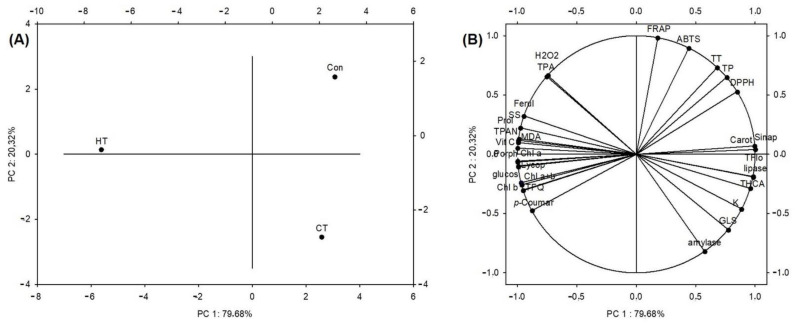
Diagram (biplot) of the Principal Component Analysis (PCA) between the measured total and individual bioactive compounds of control and treated groups of plants, their pigments, oxidative stress parameters, antioxidant potential and ability to inhibit the enzymes α-amylase, α-glucosidase and lipase. (**A**) Grouping of samples; (**B**) grouping of analyzed parameters. HT = plants watered with high-temperature water, CT = plants watered with cold water, Con = plants watered with room-temperature water, TPAN = total proanthocyanins, TPA = total phenolic acids, THCA = total hydroxycinnamic acids, SS = soluble sugars, GLS = total glucosinolates, Chl a = chlorophyll *a*, Chl b = chlorophyll *b*, Lycop = lycopene, Carot = carotenoids, Porph = porphyrins, H_2_O_2_ = hydrogen peroxide, MDA = malondialdehyde, Prol = proline, ABTS = antioxidant capacity measured by the method ABTS, FRAP = antioxidant capacity measured by the FRAP method, DPPH = antioxidant capacity measured by the DPPH method, amylase = ability to inhibit the enzyme α-amylase, lipase = ability to inhibit the enzyme lipase, glucos = ability to inhibit the enzyme α-glucosidase, Vit C = vitamin C, *p*-Coumar = *p*-coumaric acid, Ferul = ferulic acid, Sinap = sinapic acid, Q = quercetin, K = kaempferol.

Two principal components (PC 1 and PC 2) explained 100% of the variability; PC1 explained 79.68% and PC2 explained 20.32% (Figure 3). The analysis clearly separated the three analyzed groups of plants (Figure 3A). With respect to PC 1, the hot-water-treated plants separated from the other two groups. With respect to PC 2, the cold-water-treated plants were further away from the control group than the hot-water-treated group. Figure 3B shows that chlorophylls, lycopene, porphyrins, vitamin C, phenolic compounds and sugars, proline, hydrogen peroxide and malondialdehyde, and the ability to inhibit the activity of α-glucosidase contributed dominantly to the separation of the hot-water-treated group. The ability to inhibit the activity of α-amylase and lipase enzymes, glucosinolates, hydroxycinnamic acids and flavonols dominantly contributed to the separation of the group treated with cold water. Finally, total phenolics, tannins and antioxidant potential (measured by any of the three methods) contributed the most to the separation of the control group from the test groups.

#### 2.8.2. Hierarchical Clustering

**Figure 4:**
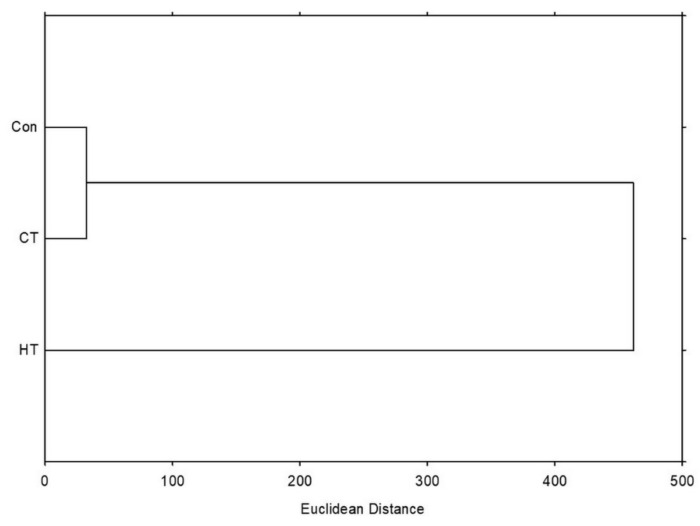
Hierarchical clustering of the control and treated groups of plants expressed as Euclidean distance, based on the measured total and individual bioactive compounds, their pigments, oxidative stress parameters, antioxidant potential and ability to inhibit enzymes α-amylase, α-glucosidase and lipase. HT = plants watered with high-temperature water, CT = plants watered with cold water, Con = plants watered with room-temperature water.

The hierarchical clustering method showed a greater similarity between the control group and the group treated with cold water, while the group treated with hot water separated more significantly (Figure 4). This was in accordance with the results of the PCA shown on Figure 3, and shows that the hot-water treatment changed the broccoli more significantly than the cold-water treatment, based on the measured variables.

#### 2.8.3. Pearson’s Correlation Coefficients

The values of Pearson’s correlation coefficients between phytochemical and oxidative stress parameters and biological activity of extracts are shown in Table 5. A very high positive correlation was noticeable between the antioxidant activity measured by the ABTS and DPPH methods with total phenolics and tannins in broccoli. Similarly, as in our work, a positive correlation between antioxidant properties and phenolic content had already been recorded for lignin samples isolated from energy crops and agro-industrial byproducts [95].

Hydrogen peroxide levels almost 100% positively correlated with total phenolic acids, and also very highly positively correlated with ferulic acid. On the contrary, almost 100% negatively correlated with total glucosinolates and very highly negatively with total hydroxycinnamic acids and kaempferol. Proline almost 100% positively correlated with total proanthocyanins and soluble sugars, and also very highly positively with total flavonoids, chlorophylls, lycopene, porphyrines, vitamin C, ferulic acid and quercetin. On the other hand, it correlated very highly negatively with total flavonols, hydroxycinnamic acids, carotenoids, sinapic acid and kaempferol. Malondialdehyde almost 100% positively correlated with total proanthocyanidins, proline and vitamin C, and also very highly positively with total flavonoids, soluble sugars, chlorophylls, lycopene, porphyrins, ferulic acid and quercetin. Almost 100% negatively correlated with total flavonols, and very highly negatively with total hydroxycinnamic acids, carotenoids, sinapic acid and kaempferol.

Regarding the ability to inhibit α-amylase enzyme activity, we recorded a very high positive correlation with total glucosinolates.

Inhibition of α-glucosidase activity very highly positively correlated with the highest number of measured parameters, with total flavonoids, proanthocyanins, soluble sugars, photosynthetic pigments, vitamin C, *p*-coumaric and ferulic acid, quercetin, proline and malondialdehyde.

Inhibition of lipase enzyme activity correlated almost 100% positively with total flavonols in broccoli. Likewise, the inhibition of this enzyme correlated very highly positively with total hydroxycinnamic acids and carotenoids, sinapic acid and kaempferol.

## 3. Materials and Methods

### 3.1. Plant Material

Broccoli seeds (*Brassica oleracea* L. convar. *botrytis* (L.) Alef. var. *cymosa* Duch.) were purchased from International Seeds Processing (ISP) GmbH (Quedlinburg, Germany) and plants were grown on a sterile substrate in pots to the stage with 2 true leaves. After that, the plants were subjected to ice water treatment (10 pieces of ice cubes, each with diameter of 2 cm, were applied daily to the substrate in the pots in which the broccoli were grown and were left until they melted) and hot-water treatment (the substrate was watered daily with the same volume of water at a temperature of 80 °C) until the stage of development with 6–8 true leaves, which was 12–14 days. The control group of plants was treated with the same volume of water at room temperature. After collecting the aerial part of a plant, it was immediately frozen in liquid nitrogen and then lyophilized. After lyophilization, the plant material was homogenized to a powder level and extracts of different concentrations were prepared in different solvents, depending on the analysis method; concentrations and solvents are listed later in each of the methods. The material included three biological replicates and three technical replicates were weighed from each biological replicate.

### 3.2. Determination of the Proportion of Total Phenols

To determine the proportion of total phenolic compounds, we prepared extracts with a concentration of 20 mg/mL in 70% ethanol and applied a method with Folin–Ciocalteu reagent [96]. The absorbance was measured on an optical microplate reader (Fluostar Optima) at a wavelength of 765 nm. Gallic acid concentration in the range of 0.5–2.5 mg/mL was used to prepare the calibration line. The results are presented as milligrams of gallic acid equivalents per gram of dry weight (mg GAE/g dw) of the sample.

### 3.3. Determination of the Proportion of Total Flavonoids

The content of total flavonoids was determined according to Zhishen et al. (1999) [97], with slight modifications. To 100 μL of extract, a volume of 400 μL of deionized water and 30 μL of NaNO_2_ (5%) was added. After 5 min incubation at room temperature, a volume of 30 μL of AlCl_3_ (10%) was added and the mixture was incubated at room temperature for an additional 6 min. Then, a volume of 200 μL of NaOH (1M) was added, and made up to a volume of 1 milliliter with deionized water. The absorbance of the reaction mixture was read at 520 nm. A quercetin solution with a concentration in the range of 0.00625–0.5 mg/mL was used to prepare the calibration line. The results are presented as milligrams of quercetin equivalents per gram of dry weight (mg QE/g dw) of the sample.

### 3.4. Determination of the Proportion of Total Proanthocyanins

Extracts with a concentration of 20 mg/mL were prepared in 70% ethanol and analyzed for the presence of total proanthocyanins as previously described [49]. The absorbance was measured on a microplate reader at a wavelength of 500 nm. A catechin solution with a concentration in the range of 0.025–1 mg/mL was used to prepare the calibration line. The results are presented as milligrams of catechin equivalents per gram of dry weight (mg CatE/g dw) of the sample.

### 3.5. Determination of the Proportion of Total Tannins

Total tannins were analyzed as in our previous work [49]. The absorbance was measured on a Nanodrop 2000c spectrophotometer at a wavelength of 700 nm. Catechin solutions with concentrations in the range of 0.00625–0.5 mg/mL were used to prepare the calibration line. The results are presented as milligrams of catechin equivalents per gram of dry weight (mg CatE/g dw) of the sample.

### 3.6. Determination of the Proportion of Total Phenolic Acids

Total phenolic acids were analyzed according to a previously published method [98]. Absorbance was measured on a microplate reader at wavelengths of 495 nm. Caffeic acid concentration in the range of 0.025–1 mg/mL was used to prepare the calibration line. The results are presented as milligrams of caffeic acid equivalents per gram of dry weight (mg CAE/g dw) of the sample.

### 3.7. Determination of the Proportion of Total Hydroxycinnamic Acids and Flavonols

Total hydroxycinnamic acids and flavonols were analyzed as in our previous work [96]. Absorbance was measured on a Nanodrop 2000c spectrophotometer at wavelengths of 320 nm for hydroxycinnamic acids and 360 nm for flavonols. A solution of caffeic acid with a concentration in the range of 0.05–0.7 mg/mL for hydroxycinnamic acids, and a solution of quercetin with a concentration of 0.00626–0.1 mg/mL for flavonols was used to prepare the calibration line. The results are presented as milligrams of caffeic acid equivalents per gram of dry weight (mg CAE/g dw) of the sample for hydroxycinnamic acids and as milligrams of quercetin equivalents per gram of dry weight (mg QE/g dw) of the sample for flavonols.

### 3.8. Determination of the Proportion of Soluble Sugars

Soluble sugars were determined as in our previous work [49]. Absorbance was measured on a microplate reader at wavelengths of 485 nm. Sucrose solution with a concentration in the range of 0.0390625–10 mg/mL was used to prepare the calibration line. The results are presented as milligrams of sucrose equivalents per gram of dry weight (mg SucE/g dw) of the sample.

### 3.9. Determination of the Proportion of Total Intact Glucosinolates

To determine the proportion of total intact glucosinolates, we prepared the extracts in hot 70% methanol and applied the already described method [99]. The absorbance was measured on a microplate reader at a wavelength of 425 nm. An aqueous solution of sinigrin in the concentration range of 0.01–0.10 mg/mL was used as a standard. The results are presented as milligrams of sinigrin equivalents per gram of dry weight (mg SinE/g dw) of the sample.

### 3.10. Determination of Parameters of Oxidative Stress

For the estimation of the level of lipid peroxidation, the method by Linić et al. (2021) was used [100]. Color intensity was quantified by measuring the absorbance at 532 and 600 nm on a MultiSkan SkyHigh Microplate Spectrophotometer (Thermo Fisher Scientific, Waltham, MA, USA). MDA content in the samples was calculated based on the molar extinction coefficient of 155 mM^−1^ cm^−1^ adapted for the measurement using the microplate reader and expressed as ng of MDA per g of dry weight (dw).

Hydrogen peroxide (H_2_O_2_) content was determined as described in Junglee et al. (2014) [101]. The color intensity was quantified by measuring the absorbance at 390 nm on a MultiSkan SkyHigh Microplate Spectrophotometer (Thermo Fisher Scientific, Waltham, MA, USA). Hydrogen peroxide content in the samples was calculated indirectly based on the calibration curve of standard H_2_O_2_ solutions of known concentrations (2475.27–61.91 μM). The results were expressed as ng of H_2_O_2_ per g of dw.

Proline content was evaluated using a method according to Ljubej et al. (2021) [47]. The color intensity was quantified by measuring the absorbance at 520 nm on a FLUOstar Optima microplate reader (BMG LABTECH, Ortenberg, Germany). The proline content in the samples was calculated indirectly based on the calibration curve of standard *L*-proline solutions of known concentrations (1.25–0.009 mg/mL). The results were expressed as mg of *L*-proline per g of dw.

### 3.11. Determination of the Proportion of Chlorophyll, Carotenoids and Porphyrins

The concentration of pigments was determined according to the method of Sumanta et al. (2014) [102]. We prepared an extract with a concentration of 15 mg/mL in 80% acetone. The mixture was mixed on a vortex mixer and then centrifuged at a temperature of 4 °C at 13,000× *g* rpm for 5 min. We separated the supernatant in a test tube, and the precipitate was extracted two more times with 80% acetone. The absorbances on a spectrophotometer (Thermo Scientific Nanodrop 2000c) at wavelengths of 470, 575, 590, 628, 647 and 663 nm were measured.

### 3.12. In Vitro Simulated Human Digestion of Extracts

The in vitro model of human digestion was performed as in our previous work (Šola et al., 2020a) [27]. The model mimics three phases of digestive process in humans; namely, mouth, stomach and small intestine. After digestion, samples were centrifuged at 11,000× *g* rpm for 10 min at 4 °C and supernatants were stored at −20 °C until further analyses.

### 3.13. Separation, Identification and Quantification of Individual Compounds by the HPLC Method

Separation, identification and quantification of individual compounds was carried out on an Agilent 1100 Series device with a UV/VIS detector, non-polar Poroshell 120 SB-C18 column 4.6 × 75 mm with a particle size of 2.7 μm, and pre-column Zorbax Rx-C18 4.6 × 12.5 mm with a particle size of 5 μm. Mobile phase A was 0.2% acetic acid (acetic acid:H_2_O; 0.2:99.8; *v*/*v*), and mobile phase B was 0.2% acetic acid and 80% methanol (acetic acid: MeOH:H_2_O; 0.2:80:19.8; *v*/*v*). The solvent gradient profile was as follows: at 0 min = 100/0, at 42 min = 20/80, at 43 min = 0/100, at 45 min = 0/100, at 45.1 min = 100/0. The flow rate was 1 mL/min and the injected volume of the samples was 25 μL. Flavonoids were analyzed at a wavelength of 360 nm, phenolic acids at 310 nm and *L*-ascorbic acid at 254 nm. We identified the compounds by comparing the retention times of the peaks obtained from the analysis of the extracts with the retention times of the peaks obtained from the analysis of the standards. Quantification of the compounds was carried out using the calibration curves of the corresponding standards.

### 3.14. Determination of Antioxidant Capacity

The antioxidant capacity was determined using three standard methods, ABTS, FRAP and DPPH, adapted to small volumes, as described in Šola et al. (2020b) [96]. An aqueous solution of Trolox in the concentration range of 0.04–0.60 mg/mL was used to create the calibration line. The results are presented as equivalents of Trolox.

### 3.15. Determination of the Effect of Broccoli Extracts on α-Amylase, α-Glucosidase and Lipase Enzyme Activity

The inhibition of α-amylase enzyme activity was measured as in Šola et al. (2022) [96]. An aqueous solution of acarbose with a concentration of 20 mg/mL was used as a positive control. The solvent in which the extract was prepared, 70% ethanol, was used as a negative control. Enzyme inhibitory activity was calculated from the equation: % inhibition = 100 − [(At − Atb)/(Ac − Acb)) × 100], where At was the absorbance of the test (with amylase), Atb was the absorbance of test blank (without amylase), Ac was the absorbance of control (with amylase) and Acb was the absorbance of control blank (without amylase).

The inhibition of α-glucosidase was determined using the pre-incubation method as described in Rusak et al. (2021) [103]. An aqueous solution of acarbose with a concentration of 20 mg/mL was used as a positive control. The solvent in which the extract was prepared, 70% ethanol, was used as a negative control. Enzyme inhibitory activity was calculated as for the α-amylase.

Pancreatic lipase inhibition assay was carried out according to Spínola et al. (2019) [104]. An ethanolic solution of orlistat with a concentration of 20 mg/mL was used as a positive control. The solvent in which the extract was prepared, 70% ethanol, was used as a negative control. Enzyme inhibitory activity was calculated as for the α-amylase.

All the absorbance measurements were performed with microplate reader Fluostar Optima (BMG Labtech GmbH, Offenburg, Germany).

### 3.16. Statistical Data Processing

Data were statistically processed using the computer software STATISTICA 12.0 (Stat Soft INC., Tulsa, OK, USA). The comparison was performed using one-way analysis of variance (ANOVA) and the application of a post hoc test of multiple comparisons (Duncan’s New Multiple Range Test, DNMRT). Values that differ at the *p* ≤ 0.05 level were considered statistically significant. Principal component analysis (PCA) was used to visualize the relationship between the samples, as well as the measured parameters. For additional visualization of the grouping of individual investigated groups based on the measured parameters, the hierarchical clustering (HC) method was used, which uses the Euclidean distance as a measure of similarity or dissimilarity between samples. To analyze the correlation between the measured parameters, the values of the Pearson’s correlation coefficients were calculated. Correlation coefficient values of 0.60–0.79 indicate a high degree of correlation, and correlation coefficient values of 0.80–1.00 indicate a very high degree of correlation [105].

## 4. Conclusions

Hot- and cold-water treatment specifically affected young broccoli. Hot water changed more variables (72%) of young broccoli than cold water (24%) treatment. Among the changed variables upon hot-water treatment, 62% increased and 38% decreased, while upon cold-water stress, 43% were increased and 57% decreased. The effect on total glucosinolates was exactly the opposite; hot water significantly decreased the proportion of total glucosinolates, while cold water increased it. Hot water also increased the concentration of total proanthocyanins, vitamin C, hydrogen peroxide, proline, malondialdehyde, chlorophyll b, lycopene and porphyrins in young broccoli. On the other hand, it lowered the concentration of carotenoids. Sinapic acid in broccoli was extremely sensitive to stress caused by hot water and its concentration decreased almost by half. Kaempferol and quercetin were oppositely affected by hot water; the first was decreased, while the latter increased. Total glucosinolates and soluble sugars were affected by hot and cold water in an opposite way; hot water decreased glucosinolates and increased soluble sugars, while cold water increased glucosinolates and decreased soluble sugars. Therefore, glucosinolates and soluble sugars could be used as biomarkers of hot/cold-water stress in broccoli. Hot-water-stressed broccoli presented a better source of *p*-coumaric and ferulic acid both before and after in vitro simulated human digestion system than broccoli treated with room-temperature water. Regarding the effect on biological activity of broccoli extracts, those of hot-water-stressed plants were significantly more efficient in the inhibition of α-glucosidase, while those of cold-water-stressed broccoli were more efficient in the inhibition of α-amylase than control plants. Among the bioactive compounds and oxidative stress parameters, the variable that was most significantly affected was proline, which was 2.5 times higher after hot-water treatment. Among the variables presenting biological activity of the extract, the most significantly affected was α-amylase inhibition; with cold-water treatment, it was 1.5 times more inhibited than with the extract of the control group. Furthermore, detailed research into the possibility of broccoli adaptation to temperature stress is highly crucial.

## Figures and Tables

**Table 1:** Effects of hot- and cold-water stress on different groups of phenolic compounds, soluble sugars and glucosinolates in broccoli.

	Con	HT	CT
TP (mg GAE/g dw)	10.56 ± 0.55 a	10.05 ± 0.98 a	10.20 ± 1.36 a
TF (mg QE/g dw)	16.31 ± 0.90 a	17.01 ± 0.87 a	16.56 ± 0.73 a
TFlo (mg QE/g dw)	0.14 ± 0.01 a	0.12 ± 0.01 b	0.14 ± 0.00 a
TPAN (mg CatE/g dw)	0.78 ± 0.05 b	1.02 ± 0.06 a	0.76 ± 0.09 b
TT (mg CatE/g dw)	5.05 ± 0.12 a	4.62 ± 0.20 a	4.70 ± 0.78 a
TPA (mg CAE/g dw)	2.23 ± 0.36 a	2.32 ± 0.26 a	2.08 ± 0.30 a
THCA (mg CAE/g dw)	0.13 ± 0.02 a	0.10 ± 0.01 b	0.14 ± 0.01 a
SS (mg SucE/g dw)	18.06 ± 0.75 b	31.14 ± 1.47 a	15.07 ± 1.61 c
GLS (mg SinE/g dw)	28.42 ± 2.76 b	24.07 ± 1.34 c	34.95 ± 2.37 a

Values represent mean ± standard deviation of three biological and three technical replicates (N = 9). Different letters indicate a significant difference among the values in a row (ANOVA, Duncan test, *p* ≤ 0.05). CatE = catechin equivalent, CAE = caffeic acid equivalent, GAE = gallic acid equivalent, CE = caffeic acid equivalent, QE = quercetin equivalent, SucE = sucrose equivalent, SinE = sinigrin equivalent, Con = plants watered with room temperature water, HT = plants watered with high-temperature water, CT = plants watered with cold water, TP = total phenolics, TF = total flavonoids, TFlo = total flavonols, TPAN = total proanthocyanins, TT = total tannins, TPA = total phenolic acids, THCA = total hydroxycinnamic acids, SS = soluble sugars, GLS = total glucosinolates.

**Table 2:** Effects of hot- and cold-water stress on parameters of oxidative stress and pigments in broccoli.

	Con	HT	CT
Hydrogen peroxide (μM/g dw)	2.33 ± 0.13 b	2.56 ± 0.30 a	1.93 ± 0.12 c
Proline (mg/g dw)	1.34 ± 0.17 b	3.31 ± 0.09 a	1.15 ± 0.12 c
Lipid peroxidation (μM MDA/g dw)	23.92 ± 0.74 b	30.62 ± 1.39 a	23.53 ± 1.91 b
Chlorophyll *a* (mg/g dw)	5.06 ± 0.24 a	5.14 ± 0.23 a	5.07 ± 0.28 a
Chlorophyll *b* (mg/g dw)	4.55 ± 0.22 b	5.35 ± 0.34 a	4.80 ± 0.63 b
Chlorophyll *a* + *b* (mg/g dw)	9.61 ± 0.28 b	10.49 ± 0.37 a	9.87 ± 0.75 b
Lycopene (mg/g dw)	0.26 ± 0.01 b	0.28 ± 0.02 a	0.26 ± 0.02 b
Carotenoids (mg/g dw)	0.63 ± 0.10 a	0.40 ± 0.19 b	0.60 ± 0.22 a
Porphyrins (mg/g dw)	25.37 ± 0.90 b	28.27 ± 1.09 a	25.71 ± 2.80 b

Values represent mean ± standard deviation of three biological and three technical replicates (N = 9). Different letters indicate a significant difference among the values in a row (ANOVA, Duncan test, *p* ≤ 0.05). Con = plants watered with room-temperature water, HT = plants watered with high-temperature water, CT = plants watered with cold water, MDA = malondialdehyde.

**Table 3:** Effects of hot- and cold-water stress on the concentration of individual phenolic compounds and vitamin C in broccoli. The results are expressed in μg/g of dry weight.

	Con	HT	CT
*p*-Coumaric acid	14.22 ± 0.68 c	19.04 ± 0.29 a	16.72 ± 0.51 b
Ferulic acid	49.41 ± 4.81 b	64.99 ± 2.62 a	43.18 ± 3.28 b
Sinapic acid	627.59 ± 37.52 a	399.80 ± 37.67 b	599.33 ± 10.14 a
Total identified phenolic acids	691.22 ± 42.02 a	423.83 ± 35.48 b	659.24 ± 7.85 a
Kaempferol	155.82 ± 2.24 a	140.72 ± 3.81 b	166.71 ± 6.38 a
Quercetin	32.93 ± 0.49 b	35.11 ± 0.73 a	33.70 ± 0.13 b
Isorhamnetin	38.54 ± 5.45 a	38.14 ± 2.64 a	38.06 ± 5.52 a
Total identified flavonols	227.29 ± 5.66 ab	213.98 ± 6.44 b	238.47 ± 7.52 a
Luteolin	27.13 ± 0.56 a	27.26 ± 0.12 a	26.54 ± 0.20 a
Total identified flavonoids	254.41 ± 5.11 ab	241.23 ± 6.41 b	265.00 ± 7.56 a
Total identified phenolics	945.63 ± 39.85 a	665.07 ± 29.74 b	924.24 ± 8.27 a
*L*-Ascorbic acid	1144.43 ± 66.64 b	1523.00 ± 70.30 a	1143.09 ± 33.32 b
Total identified compounds	2090.06 ± 103.54 a	2188.06 ± 88.29 a	2067.34 ± 39.40 a

Values represent mean ± standard deviation of three biological replicates. Different letters indicate a significant difference among the values in a row (ANOVA, Duncan test, *p* ≤ 0.05). Con = plants watered with room-temperature water, HT = plants watered with high-temperature water, CT = plants watered with cold water.

**Table 4:** Effects of hot- and cold-water stress on antioxidant potential of broccoli extract. The results are expressed in trolox equivalent.

	Con	HT	CT
ABTS	9.56 ± 0.72 a	8.72 ± 0.85 a	8.60 ± 0.83 a
FRAP	12.74 ± 0.61 a	12.50 ± 0.46 a	12.35 ± 0.62 a
DPPH	4.72 ± 0.40 a	3.69 ± 0.46 b	4.13 ± 0.51 b

Values represent mean ± standard deviation of three biological and three technical replicates (N = 9). Different letters indicate a significant difference among the values in a row (ANOVA, Duncan test, *p* ≤ 0.05). Con = plants watered with room-temperature water, HT = plants watered with high-temperature water, CT = plants watered with cold water.

**Table 5:** Pearson’s correlation coefficients between the measured variables of young broccoli plants.

	**TP**	**TF**	**TFlo**	**TT**	**TPAN**	**TPA**	**THCA**	**SS**	**GLS**	**Chl a**	**Chl b**	**Chl a + b**	**Lycop**	**Carot**	**Porph**	**Vit C**	***p*** **-Coumar**	**Ferul**	**Sinap**	**Q**	**K**	**ABTS**	**FRAP**	**DPPH**	**H_2_O_2_**	**Prol**	**MDA**	**α** **-amylase**	**α** **-glucos**	**Lipase**
TP	1.00																													
TF	**−0.92**	1.00																												
TFlo	0.62	−0.88	1.00																											
TT	**0.99**	−0.87	0.53	1.00																										
TPAN	−0.68	**0.91**	−1.00	−0.59	**1.00**																									
TPA	−0.15	0.52	−0.86	−0.03	0.83	1.00																								
THCA	0.54	−0.82	**0.99**	0.44	**−0.99**	**−0.91**	1.00																							
SS	−0.60	0.86	**−1.00**	−0.50	**0.99**	0.88	**−1.00**	1.00																						
GLS	0.17	−0.54	0.88	0.06	−0.84	**−1.00**	**0.92**	−0.89	1.00																					
Chl a	−0.80	**0.97**	**−0.97**	−0.73	**0.98**	0.71	**−0.94**	**0.96**	−0.73	1.00																				
Chl b	**−0.90**	**1.00**	**−0.90**	−0.85	**0.93**	0.56	−0.85	0.88	−0.58	**0.98**	1.00																			
Chl a + b	−0.89	**1.00**	**−0.91**	−0.84	**0.94**	0.57	−0.86	0.89	−0.60	**0.98**	**1.00**	1.00																		
Lycop	−0.82	**0.98**	**−0.96**	−0.75	**0.98**	0.69	**−0.92**	**0.95**	−0.71	**1.00**	**0.99**	**0.99**	1.00																	
Carot	0.80	**−0.97**	**0.97**	0.73	**−0.98**	−0.71	**0.93**	**−0.96**	0.73	**−1.00**	**−0.98**	**−0.99**	**−1.00**	1.00																
Porph	−0.80	**0.97**	**−0.97**	−0.72	**0.98**	0.71	**−0.94**	**0.96**	−0.73	**1.00**	**0.98**	**0.98**	**1.00**	**−1.00**	1.00															
Vit C	−0.72	**0.93**	**−0.99**	−0.64	**1.00**	0.79	**−0.97**	**0.99**	−0.80	**0.99**	**0.95**	**0.96**	**0.99**	**−0.99**	**0.99**	1.00														
*p*-Coumar	**−0.98**	**0.98**	−0.77	−0.95	0.82	0.35	−0.71	0.75	−0.38	**0.91**	**0.97**	**0.97**	**0.92**	**−0.91**	**0.91**	0.85	1.00													
Ferul	−0.51	0.80	**−0.99**	−0.41	**0.98**	**0.93**	**−1.00**	**0.99**	−0.94	**0.92**	0.83	0.84	**0.91**	**−0.92**	**0.93**	**0.96**	0.68	1.00												
Sinap	0.79	**−0.96**	**0.97**	0.71	**−0.99**	−0.73	**0.95**	**−0.97**	0.75	**−1.00**	**−0.98**	**−0.98**	**−1.00**	**1.00**	**−1.00**	**−1.00**	−0.90	**−0.93**	1.00											
Q	**−0.92**	**1.00**	−0.88	−0.87	**0.91**	0.52	−0.83	0.86	−0.54	**0.97**	**1.00**	**1.00**	**0.98**	**−0.97**	**0.97**	**0.94**	**0.98**	0.80	**−0.96**	1.00										
K	0.37	−0.70	**0.96**	0.27	**−0.94**	**−0.97**	**0.98**	**−0.97**	**0.98**	−0.85	−0.74	−0.75	−0.84	0.85	−0.86	**−0.91**	−0.56	**−0.99**	0.87	−0.71	1.00									
ABTS	**0.92**	−0.70	0.27	**0.96**	−0.33	0.26	0.17	−0.23	−0.23	−0.50	−0.66	−0.64	−0.53	0.50	−0.49	−0.39	−0.82	−0.13	0.48	−0.69	−0.02	1.00								
FRAP	0.78	−0.47	−0.01	0.84	−0.06	0.51	−0.11	0.04	−0.48	−0.24	−0.43	−0.41	−0.28	0.25	−0.24	−0.13	−0.63	0.15	0.22	−0.47	−0.29	**0.96**	1.00							
DPPH	**0.99**	**−0.97**	0.73	**0.97**	−0.78	−0.29	0.66	−0.71	0.32	−0.88	**−0.96**	**−0.95**	−0.90	0.88	−0.88	−0.82	**−1.00**	−0.63	0.87	−0.97	0.51	0.85	0.67	1.00						
H_2_O_2_	−0.13	0.51	−0.86	−0.02	0.82	**1.00**	**−0.91**	0.88	**−1.00**	0.70	0.55	0.57	0.68	−0.70	0.71	0.78	0.34	**0.92**	−0.72	0.51	**−0.97**	0.27	0.52	−0.28	1.00					
Prol	−0.67	**0.90**	**−1.00**	−0.58	**1.00**	0.83	**−0.99**	**1.00**	−0.85	**0.98**	**0.92**	**0.93**	**0.97**	**−0.98**	**0.98**	**1.00**	0.81	**0.98**	**−0.99**	**0.91**	**−0.94**	−0.32	−0.05	−0.77	0.83	1.00				
MDA	−0.69	**0.92**	**−1.00**	−0.61	**1.00**	0.82	**−0.98**	**0.99**	−0.83	**0.99**	**0.94**	**0.94**	**0.98**	**−0.99**	**0.99**	**1.00**	0.83	**0.97**	**−0.99**	**0.92**	**−0.93**	−0.35	−0.08	−0.79	0.81	**1.00**	1.00			
α-amylase	−0.10	−0.30	0.72	−0.21	−0.67	**−0.97**	0.79	−0.74	**0.96**	−0.52	−0.34	−0.36	−0.49	0.51	−0.53	−0.62	−0.12	−0.81	0.54	−0.30	0.89	−0.48	−0.70	0.05	−0.97	−0.68	−0.65	1.00		
α-glucos	−0.82	**0.98**	**−0.96**	−0.75	**0.98**	0.68	**−0.92**	**0.95**	−0.70	**1.00**	**0.99**	**0.99**	**1.00**	**−1.00**	**1.00**	**0.99**	**0.92**	**0.91**	**−1.00**	**0.98**	−0.83	−0.53	−0.28	−0.90	0.67	**0.97**	**0.98**	−0.49	1.00	
lipase	0.62	−0.88	**1.00**	0.53	**−1.00**	−0.87	**0.99**	**−1.00**	0.88	**−0.97**	−0.90	**−0.91**	**−0.96**	**0.97**	**−0.97**	**−0.99**	−0.77	**−0.99**	**0.97**	−0.88	**0.96**	0.26	−0.01	0.73	−0.86	**−1.00**	**−1.00**	0.72	**−0.96**	1.00

TPAN = total proanthocyanidins, TPA = total phenolic acids, THCA = total hydroxycinnamic acids, SS = soluble sugars, GLS = total glucosinolates, Chl a = chlorophyll *a*, Chl b = chlorophyll *b*, Lycop = lycopene, Carot = carotenoids, Porph = porphyrins, H_2_O_2_ = hydrogen peroxide, MDA = malondialdehyde, Prol = proline, ABTS = antioxidant capacity measured by the ABTS method, FRAP = antioxidant capacity measured by the FRAP method, DPPH = antioxidant capacity measured by the DPPH method, amylase = ability to inhibit the enzyme α-amylase, lipase = ability to inhibit the enzyme lipase, glucos = ability to inhibit the enzyme α-glucosidase, Vit C = vitamin C, *p*-Coumar = *p*-coumaric acid, Ferul = ferulic acid, Sinap = sinapic acid, Q = quercetin, K = kaempferol.

## Data Availability

Data is contained within the tables, figures and Supplementary Material of the manuscript.

## Supplementary Materials

The following supporting information can be downloaded at: https://www.mdpi.com/article/10.3390/plants12051135/s1, Figure S1: Representation of decreased, unchanged and increased variables in young broccoli plants due to hot/cold-water treatment. Decreased/increased value = there was a statistically significant decrease/increase in value compared to the control group of plants watered with room-temperature water. No change = no statistically significant change in values compared to the control group of plants, *p* ≤ 0.05; Figure S2: Proportion of each identified compound in predigested extracts of (A) control, (B) group treated with hot water and (C) group treated with cold water; Figure S3: Intensity and direction of the influence of HT and CT stress on the concentration of (A) different groups of phenolic compounds, (B) sugars, total glucosinolates and pigments and (C) individual compounds compared to the control group. GLS = total glucosinolates, HT = high temperature, CT = cold temperature; Figure S4: Intensity and direction of the influence of HT and CT stress on the (A) antioxidant potential, (B) parameters of oxidative stress and (C) effect of broccoli extracts on the activity of enzymes compared to the control group. H_2_O_2_ = hydrogen peroxide, Prol = proline, MDA = malondialdehyde; Table S1: Effect of hot and cold water on the bioavailability (%) of individual phenolic compounds and vitamin C in each phase of in vitro digestion of broccoli compared to the concentration in predigested extracts; Table S2: Distribution of the analyzed variables of young broccoli plants with respect to their resistance/susceptibility due to hot/cold-water treatment. Decreased/increased value = there was a statistically significant decrease/increase in value compared to the control group of plants watered with room-temperature water. Individual phenolic compounds and vitamin C refer to the extract before digestion. No change = no statistically significant change in values compared to the control group of plants, *p* ≤ 0.050; Table S3: Quality control of the HPLC analytical system.
