# Supplementary material for: Effect of Hot- and Cold-Water Treatment on Broccoli Bioactive Compounds, Oxidative Stress Parameters and Biological Effects of Their Extracts
Source: Plants (Basel). 2023 Mar 2;12(5):1135. doi: 10.3390/plants12051135 (PMC10005114; doi:10.3390/plants12051135)
Supplement: Supplementary file 1 [file plants-12-01135-s001.zip › table S1.pdf]

Table S1.

		Con	CT	HT
p-Coumaric acid	Salivary phase	107.83 ± 1.56 B a	99.93 ± 3.34 AB b	97.07 ± 2.51 A b
	Gastric phase	134.14 ± 4.47 A a	104.34 ± 2.63 A b	98.42 ± 4.72 A b
	Intestinal phase	128.94 ± 7.25 A a	97.92 ± 0.83 B b	103.92 ± 2.96 A b
Ferulic acid	Salivary phase	97.59 ± 7.59 B a	104.60 ± 5.27 B a	102.53 ± 4.73 A a
	Gastric phase	105.43 ± 6.07 B a	103.93 ± 3.99 B a	104.67 ± 2.31 A a
	Intestinal phase	121.73 ± 2.15 A a	117.76 ± 1.73 A a	100.98 ± 1.41 A b
Sinapic acid	Salivary phase	76.51 ± 5.32 B c	122.85 ± 4.84 A a	97.33 ± 5.50 A b
	Gastric phase	103.13 ± 6.06 A b	126.95 ± 7.23 A a	99.90 ± 4.18 A b
	Intestinal phase	109.14 ± 9.36 A b	131.84 ± 3.77 A a	102.61 ± 6.62 A b
Kaempferol	Salivary phase	94.75 ± 2.81 A b	101.79 ± 2.83 A a	97.53 ± 0.75 A ab
	Gastric phase	95.54 ± 2.55 A b	101.49 ± 0.91 A a	97.96 ± 2.47 A ab
	Intestinal phase	nd	nd	nd
L-ascorbic acid	Salivary phase	98.20 ± 1.47 A ab	102.55 ± 2.39 A a	95.67 ± 3.45 A b
	Gastric phase	96.20 ± 2.33 A a	98.95 ± 3.47 A a	94.06 ± 1.44 A a
	Intestinal phase	98.35 ± 5.49 A ab	105.28 ± 3.42 A a	90.98 ± 3.67 A b

Values represent mean ± standard deviation of 3 replicates.
Different capital letters indicate a significant difference among the values in a column, and different small letters indicate a significant difference among the values in a row (ANOVA, Duncan test, $p \leq 0.05$).
