# Supplementary material for: Effect of Hot- and Cold-Water Treatment on Broccoli Bioactive Compounds, Oxidative Stress Parameters and Biological Effects of Their Extracts
Source: Plants (Basel). 2023 Mar 2;12(5):1135. doi: 10.3390/plants12051135 (PMC10005114; doi:10.3390/plants12051135)
Supplement: Supplementary file 1 [file plants-12-01135-s001.zip › Table S2.pdf]

Table S2.

	Decreased value	No change	Increased value
**Hot water**	• Carotenoids<br>• Total hydroxycinnamic acids<br>• Total flavonols<br>• Total glucosinolates<br>• DPPH<br>• Lipase inhibition<br>• Sinapic acid<br>• Kaempferol	• Chlorophyll *a*<br>• Total phenolics<br>• Total flavonoids<br>• Total tannins<br>• Total phenolic acids<br>• ABTS<br>• FRAP<br>• α-amylase inhibition	• Chlorophyll *b*<br>• Lycopene<br>• Porphyrins<br>• Total proanthocyanidins<br>• Soluble sugars<br>• Vitamin C<br>• Ferulic acid<br>• *p*-Coumaric cid<br>• Quercetin<br>• α-glucosidase inhibition<br>• H_2O_2<br>• Proline<br>• Lipid peroxidation
**Cold water**	• Soluble sugars<br>• DPPH<br>• H_2O_2<br>• Proline	• Chlorophyll *a*<br>• Chlorophyll *b*<br>• Lycopene<br>• Carotenoids<br>• Porphyrins<br>• Total proanthocyanidins<br>• Total hydroxycinnamic acids<br>• Total flavonols<br>• Total phenolics<br>• Total flavonoids<br>• Total tannins<br>• Total phenolic acids<br>• Vitamin C<br>• Ferulic acid<br>• Sinapic acid<br>• Quercetin<br>• Kaempferol<br>• ABTS<br>• FRAP<br>• Lipase inhibition<br>• α-glucosidase inhibition<br>• Lipid peroxidation	• Total glucosinolates<br>• α-Amylase inhibition<br>• *p*-Coumaric acid
