# Supplementary material for: Effect of Hot- and Cold-Water Treatment on Broccoli Bioactive Compounds, Oxidative Stress Parameters and Biological Effects of Their Extracts
Source: Plants (Basel). 2023 Mar 2;12(5):1135. doi: 10.3390/plants12051135 (PMC10005114; doi:10.3390/plants12051135)
Supplement: Supplementary file 1 [file plants-12-01135-s001.zip › Table S3.pdf]

Vitamin C

Mass (µg)	Abs	Found conc (µg)	Recovery (%)
0,01	41,13	0,01	103,38
0,025	108,76	0,02	94,37
0,05	250,51	0,05	102,75
0,1	497,71	0,10	99,82
0,15	753,08	0,15	99,92
0,2	747,15	0,15	74,36
**Mean**			95,77
**SD**			10,96
**SE of Intercept**			55,29
**SD of Intercept**			135,44
**LOD**			0,06
**LOQ**			0,17

**Parameter**	**Value**
Accuracy	95,77 ± 10,96
Slope	5102,3
Intercept	-11,619
Linearity range	0,01-0,20 µg
Correlation coefficient	0,999849989
SE of intercept	55,29
SD of intercept	135,44
LOD	0,06
LOQ	0,17

***p*-Coumaric acid**

Mass (µg)	Abs	Found conc (µg)	Recovery (%)
0,01	175,16	0,02	236,67
0,025	453,44	0,06	226,96
0,05	908,12	0,11	221,55
0,1	1821,45	0,22	219,32
0,15	2762,26	0,33	220,75
0,2	3848,72	0,46	230,13
**Mean**			225,90
**SD**			6,69
**SE of Intercept**			36,56
**SD of Intercept**			89,56
**LOD**			0,03
**LOQ**			0,10

**Parameter**	**Value**
Accuracy	225,90 ± 6,69
Slope	18442
Intercept	-11,523
Linearity range	0,01-0,20 µg
Correlation coefficient	1
SE of intercept	36,56
SD of intercept	89,56
LOD	0,03
LOQ	0,10

Ferulic acid

Mass (µg)	Abs	Found conc (µg)	Recovery (%)
0,01	85,07	0,01	129,60
0,025	161,34	0,02	88,10
0,05	387,68	0,05	97,85
0,1	828,43	0,10	101,30
0,15	1236,38	0,15	99,86
0,2	2181,18	0,26	131,04
**Mean**			107,96
**SD**			17,93
**SE of Intercept**			110,88
**SD of Intercept**			271,61
**LOD**			0,11
**LOQ**			0,35

**Parameter**	**Value**
Accuracy	107,96 ± 17,93
Slope	8414,3
Intercept	-23,978
Linearity range	0,01-0,26 µg
Correlation coefficient	0,999249719
SE of intercept	110,88
SD of intercept	271,61
LOD	0,11
LOQ	0,35

Sinapic acid

Mass (µg)	Abs	Found conc (µg)	Recovery (%)
0,01	72,06	0,01	114,14
0,025	177,26	0,02	95,66
0,05	423,35	0,05	106,33
0,1	893,99	0,11	109,10
0,15	1339,13	0,16	108,00
0,2	2318,9	0,28	139,22
**Mean**			112,07
**SD**			14,62
**SE of Intercept**			109,34
**SD of Intercept**			267,84
**LOD**			0,10
**LOQ**			0,29

**Parameter**	**Value**
Accuracy	112,07 ± 14,62
Slope	9179,2
Intercept	-33,85
Linearity range	0,01-0,28 µg
Correlation coefficient	0,999699955
SE of intercept	109,34
SD of intercept	267,84
LOD	0,10
LOQ	0,29

Quercetin

Mass (µg)	Abs	Found conc (µg)	Recovery (%)
0,01	31,74	0,01	122,39
0,025	92,15	0,02	92,74
0,05	233,76	0,05	97,69
0,1	519,3	0,10	100,58
0,15	792,87	0,15	100,10
0,2	1467,88	0,27	136,23
**Mean**			108,29
**SD**			17,09
**SE of Intercept**			84,53
**SD of Intercept**			207,07
**LOD**			0,12
**LOQ**			0,38

**Parameter**	**Value**
Accuracy	108,29 ± 17,09
Slope	5519
Intercept	-35,806
Linearity range	0,01 - 0,27 µg
Correlation coefficient	0,99959992
SE of intercept	84,53
SD of intercept	207,07
LOD	0,12
LOQ	0,38

Isorhamnetin

Mass (µg)	Abs	Found conc (µg)	Recovery (%)
0,01	45,69	0,01	82,80
0,025	123,5	0,02	70,11
0,05	265,2	0,03	68,73
0,1	533,13	0,07	66,21
0,15	825,89	0,10	67,34
0,2	1170,26	0,14	70,96
**Mean**			71,02
**SD**			6,02
**SE of Intercept**			15,68
**SD of Intercept**			38,41
**LOD**			0,01
**LOQ**			0,04

**Parameter**	**Value**
Accuracy	71,02 ± 6,02
Slope	5560,4
Intercept	-13,866
Linearity range	0,01-0,20 µg
Correlation coefficient	0,999849989
SE of intercept	15,68
SD of intercept	38,41
LOD	0,01
LOQ	0,04

Kaempferol

Mass (µg)	Abs	Found conc (µg)	Recovery (%)
0,01	50,12	0,01	121,32
0,025	138,55	0,02	93,67
0,05	339,34	0,05	98,09
0,1	737,81	0,10	99,90
0,15	1134,37	0,15	100,34
0,2	1467,88	0,19	96,54
**Mean**			101,64
**SD**			9,94
**SE of Intercept**			13,71
**SD of Intercept**			33,58
**LOD**			0,01
**LOQ**			0,04

**Parameter**	**Value**
Accuracy	101,64 ± 9,94
Slope	7835,5
Intercept	-44,942
Linearity range	0,01-0,19 µg
Correlation coefficient	0,999699955
SE of intercept	13,71
SD of intercept	33,58
LOD	0,01
LOQ	0,04

Luteolin

Mass (µg)	Abs	Found conc (µg)	Recovery (%)
0,01	70,58	0,01	112,38
0,025	191,99	0,03	102,67
0,05	408,77	0,05	102,86
0,1	813,58	0,10	99,54
0,15	1241,46	0,15	100,26
0,2	1756,62	0,21	105,81
**Mean**			103,92
**SD**			4,70
**SE of Intercept**			21,59
**SD of Intercept**			52,89
**LOD**			0,02
**LOQ**			0,06

**Parameter**	**Value**
Accuracy	103,92 ± 4,70
Slope	8347,4
Intercept	-14
Linearity range	0,01-0,20 µg
Correlation coefficient	0,999949999
SE of intercept	21,59
SD of intercept	52,89
LOD	0,02
LOQ	0,06
