# Supplementary figures and images for: Effect of Hot- and Cold-Water Treatment on Broccoli Bioactive Compounds, Oxidative Stress Parameters and Biological Effects of Their Extracts
Source: Plants (Basel). 2023 Mar 2;12(5):1135. doi: 10.3390/plants12051135 (PMC10005114; doi:10.3390/plants12051135)

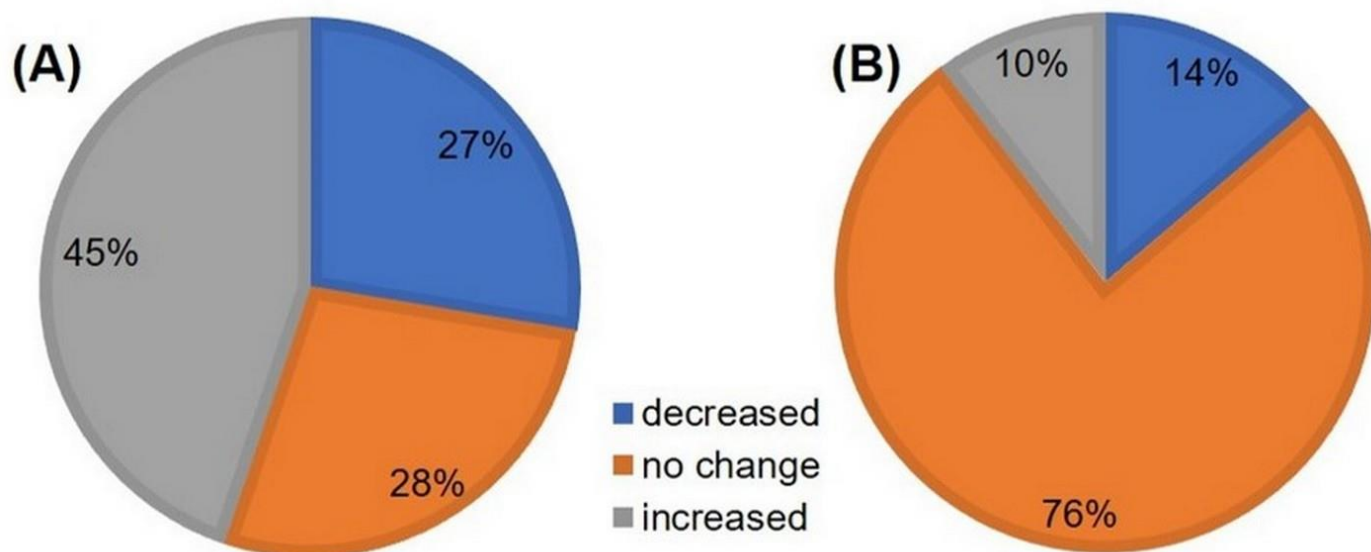

Figure S1

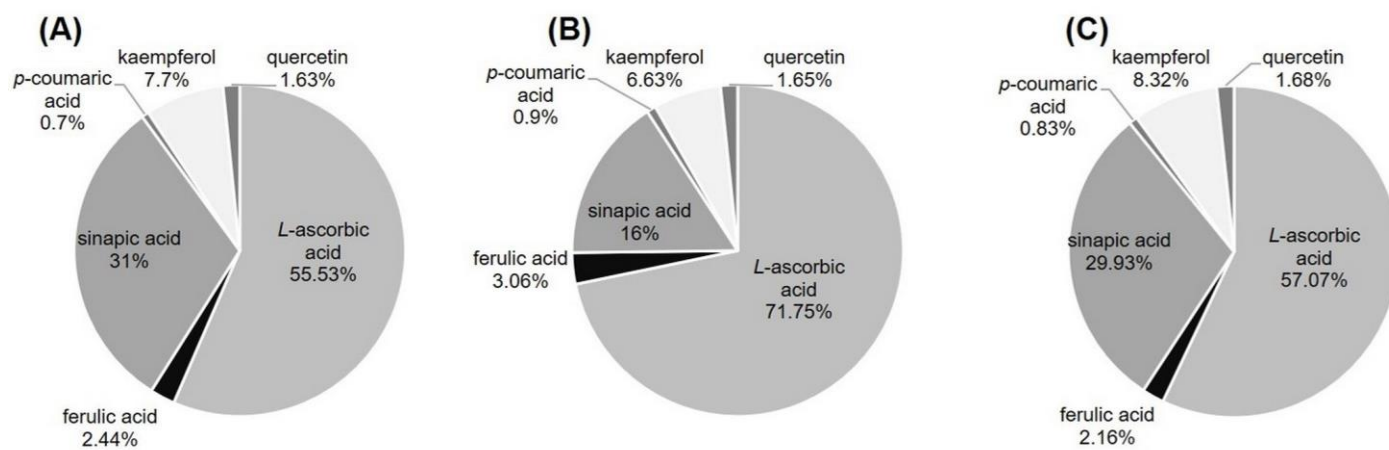

Figure S2

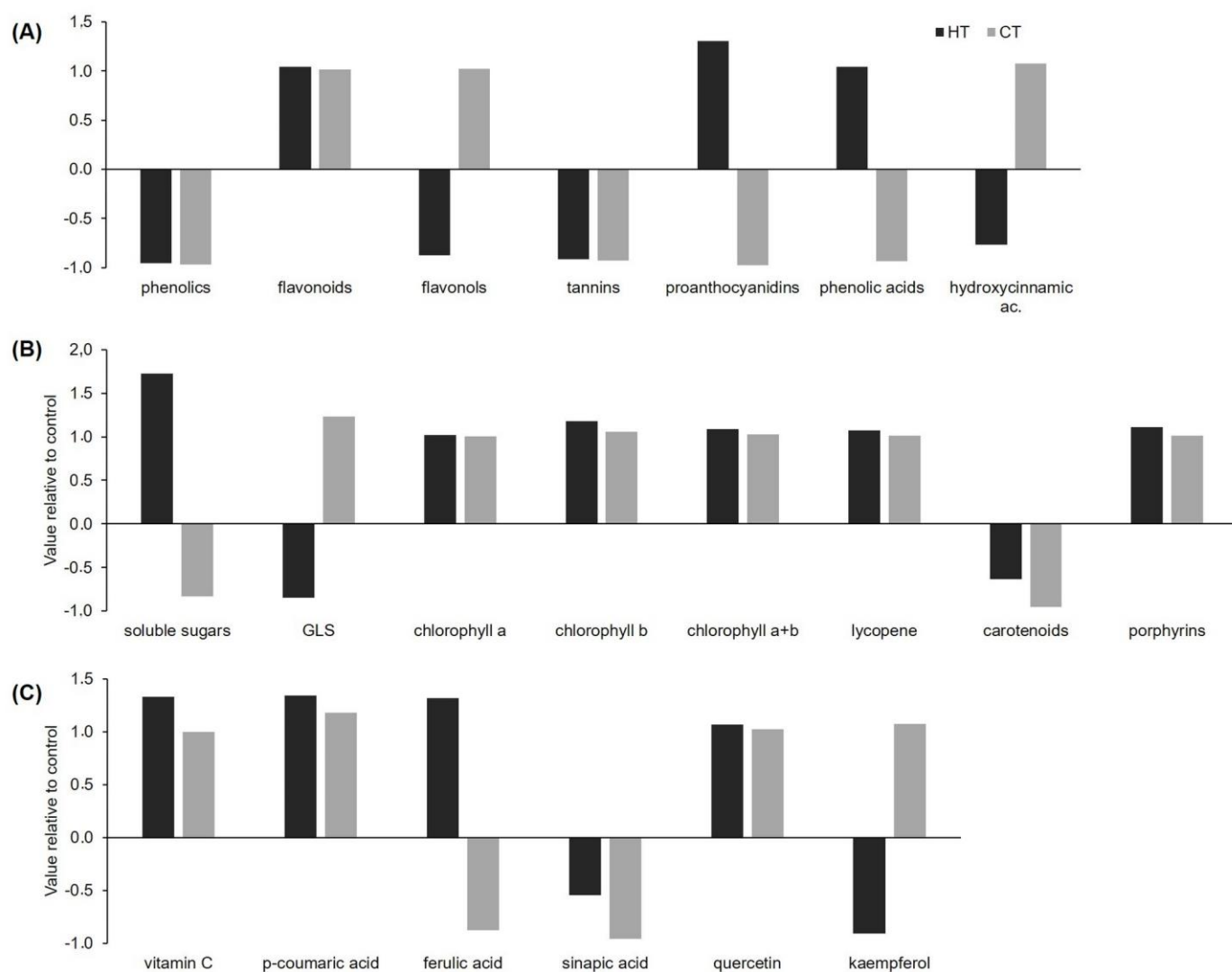

**Figure S3**

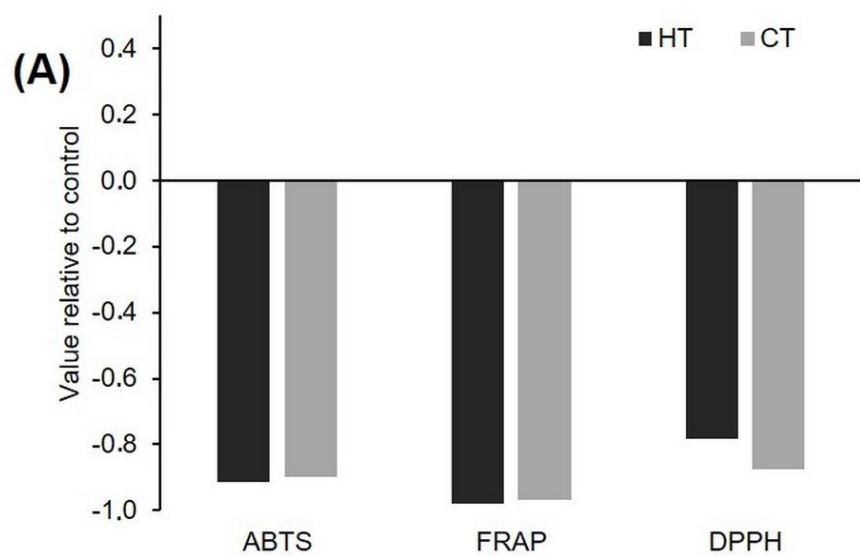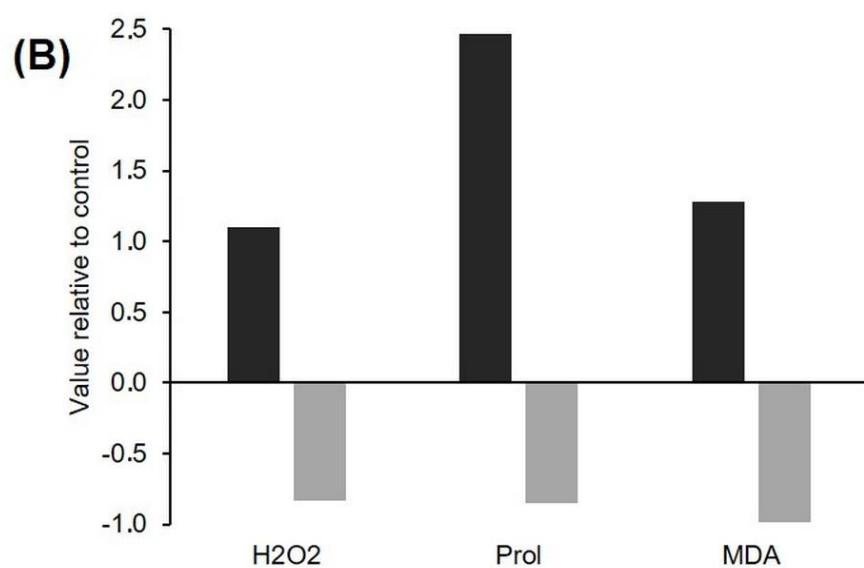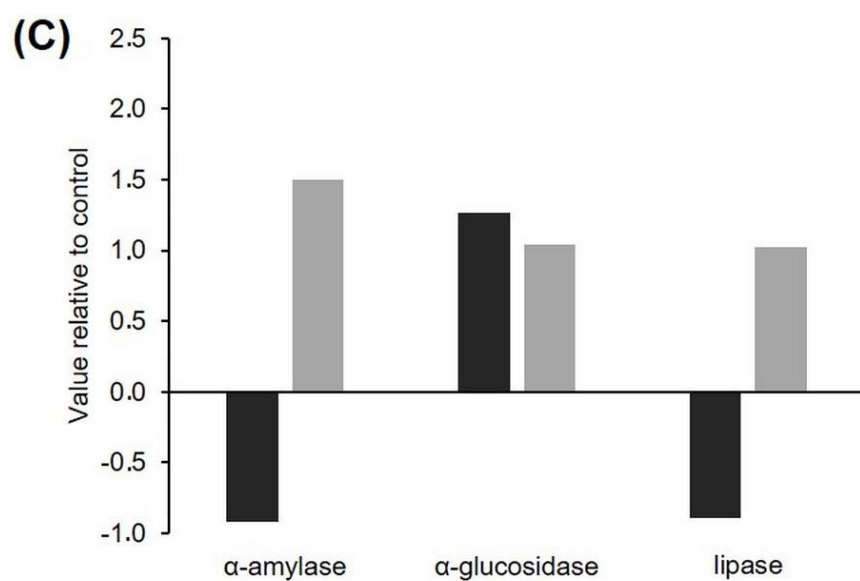

Figure S4

Supplement: Supplementary file 1 [file plants-12-01135-s001.zip › Figures S1-S4.pdf]
